# Effect of household water treatment with chlorine on diarrhea among children under the age of five years in rural areas of Dire Dawa, eastern Ethiopia: a cluster randomized controlled trial

**DOI:** 10.1186/s40249-020-00680-9

**Published:** 2020-06-08

**Authors:** Ephrem Tefera Solomon, Sirak Robele, Helmut Kloos, Bezatu Mengistie

**Affiliations:** 1 Ethiopian Institute of Water Resources, Addis Ababa University, Addis Ababa, Ethiopia; 2 University of California, San Francisco Medical Center, San Francisco, CA USA; 3 Haramaya University, College of Health and Medical Sciences, Harar, Ethiopia

**Keywords:** Diarrhea, Effect, Water treatment with chlorine, Under-five children, WaterGuard, Cluster randomized controlled trial, Ethiopia

## Abstract

**Background:**

Diarrheal disease is a leading cause of child mortality and morbidity worldwide. Household water treatment with chlorine significantly reduces morbidity due to waterborne diseases. However, the effect of point-of-use (POU) water treatment in improving the quality of water in areas where POU is not provided free of charge and the effectiveness of home visits in inspiring household members to use POU regularly have not been studied. The objective of this study was to evaluate the effectiveness of drinking water disinfection by chlorination on diarrheal disease reduction among children under the age of 5 years in rural eastern Ethiopia.

**Methods:**

A cluster randomized controlled trial was carried out in rural Dire Dawa from October 2018 through January 2019. The 405 households were randomized to intervention and control arms and intervention materials were distributed after conducting a baseline survey. This trial evaluated the effectiveness of household drinking water disinfection by chlorination in reducing incidence of diarrhea among children under the age of 5 years. Intervention households received 1.2% sodium hypochlorite with demonstration of its proper use. Participants in the control households continued with their usual habits of water collection and water storage. Generalized estimation equation (GEE) with log link Poisson distribution family and exchangeable correlation matrix was used to compute crude incidence rate ratio (IRR), adjusted IRR and the corresponding 95% confidence intervals.

**Results:**

In the intervention households, in total, 281 cases of diarrhea were documented (8.7 cases per 100 person-weeks observation); in the control households, in total 446 cases of diarrhea were documented (13.8 cases per 100 person-weeks observation). A 36.0% (adjusted IRR = 0.64, 95% *CI*: 0.57–0.73) reduction in incidence of diarrhea was observed in the intervention arm when compared with the control arm. The highest and the lowest reductions were obtained in children of age ranges 1 to 2 years and 3 to 4 years, 42.7 and 30.4%, respectively. Adherence to the intervention was 81.3% as measured by free residual chlorine test.

**Conclusions:**

In rural areas where diarrhea is the second leading cause of morbidity, water chlorination at the household level using liquid bleach considerably reduced episodes of diarrhea among children under the age of 5 years. Therefore, chlorinating drinking water at the household level may be a valuable interim solution for reducing the incidence of diarrheal diseases until potable water is made accessible to the majority of the population in Dire Dawa Administration and other Ethiopian communities.

**Trial registration:**

PACTR, PACTR201807815961394. Registered 16 July 2018, www.pactr.org

## Background

Diarrhea was responsible for an estimated 533 768 deaths among children younger than 5 years globally in 2017, a rate of 78.4 deaths per 100 000 children [1]. The problem is aggravated in children living in rural rather than urban areas [2]. A recent systematic review and meta-analysis examining the prevalence and determinants of diarrhea among under-five children in Ethiopia indicated that children from rural households were 1.9 times more likely to have diarrhea than their urban counterparts [3]. Unsafe drinking water is a major cause of diarrhea deaths and disease, especially for young children and vulnerable populations in low-income countries [4]. Furthermore, the majority of the world’s population does not have access to water piped into their homes and must carry, transport, and store water within their homes. In these situations, recontamination of drinking water is often significant and is increasingly recognized as an important public health issue [5]. Unhygienic handling of water during transport or within the home can contaminate previously safe water. WHO estimates that 94.0% of diarrhea cases are preventable through modifications to the environment, including increasing the availability of clean water and improving sanitation and hygiene [6]. Therefore, promoting household water treatment and safe storage (HWTS) helps vulnerable populations to take charge of their own water security by providing them with the knowledge and tools that enable them to treat their own drinking water [7].

Interventions to improve water quality are generally effective in preventing diarrhea and effectiveness is usually positively associated with compliance [8]. According to United Nations International Children’s Emergency Fund report, point-of-use water treatment with chlorine solution has been estimated to reduce diarrheal disease by 29.0% [9]. However, mismanagement of excess chlorine reacts with precursors in the water that forms disinfectant-by-products (DBPs), like trihalomethane (THM) and haloacetic acids (HAAs) which cause the risk of cancer [10]. Various intervention studies achieved reductions in incidence and longitudinal prevalence of diarrhea among children under 5 years by 11.0 to 90.0% [11–20]. Conversely, some interventions failed to reduce diarrhea levels [21–24].

According to Dire Dawa Regional Health Bureau, in 2016 diarrhea was the second leading cause of morbidity next to upper respiratory infections, in children under the age of 5 years, affecting 19 194 (30.8%) children (Dire Dawa Administration Regional Health Bureau 2015/2016 Budget Year Annual Report, unpublished).

Populations with microbiologically safe piped water tend to have the lowest mortality rates from diarrheal disease [25]. However, piped water supplies are still scarce in many communities in low-income countries. Thus, until these services become widely available in these countries, POU water treatment is a potential interim solution to the problems caused by diarrhea [25]. However, the effect of the POU treatment in improving water quality against post-source contamination, the magnitude of the intervention effect in areas where POU is not provided free of charge, and the effectiveness of home visits in inspiring household members to use POU regularly have not been determined. Determining these relationships may aid the effort to upscale point-of-use to a larger community level. Hence, the objective of this study was to evaluate the effectiveness of drinking water disinfection by chlorination at the household level in diarrheal disease reduction among children under the age of 5 years in rural parts of Dire Dawa, eastern Ethiopia.

## Methods

### Study area

Dire Dawa, one of the two federal cities, is a commercial and industrial center located 505 km east of Addis Ababa on the Addis Ababa–Djibouti railroad. Dire Dawa Administration consists of 9 urban and 38 rural *kebeles* (the smallest administrative units). According to the Dire Dawa Water, Mine and Energy Bureau, safe drinking water in rural areas is supplied by protected springs, protected shallow wells, and deep wells. Safe drinking water reached 71.8% of the area in 2017. Thirty-three health posts and seven health centers render health services to the rural population (Dire Dawa Administration Health Bureau 2017, 6 months report, unpublished). The projected population of Dire Dawa Administration, Ethiopia, in 2018 (the year in which the data were collected) was 479 000, of which 240 000 were males and 239 000 were females; the male to female ratio was nearly 1:1. Of these, 176 000 (36.7%) lived in rural areas and the rural population are sparsely populated [26]. Rural households had an average of 4.9 persons per household. According to the 2017 Regional Health Bureau reports, there were 34 150 households in the four districts of rural Dire Dawa with 20 118 children under the age of 5 years. The latrine coverage of the administration was 54.9%, and rural households stored their drinking water in 20 l jerry-cans (Dire Dawa Administration Regional Health Bureau: 2017 Facility Information, unpublished).

### Source and study population

The source population consisted of households with at least one child under the age of 5 years in the 38 rural *kebeles* of the four districts; the study population consisted of households with at least one child under the age of 5 years selected randomly from two *kebeles*.

### Inclusion and exclusion criteria

Households having at least one child less than 5 years of age were included. Households having mothers/caregivers who were severely ill and unable to respond to the questionnaire, households having under-five children with persistent diarrhea, and households with children younger than 6 months were excluded.

### Trial design and procedure

**Fig. 1:**
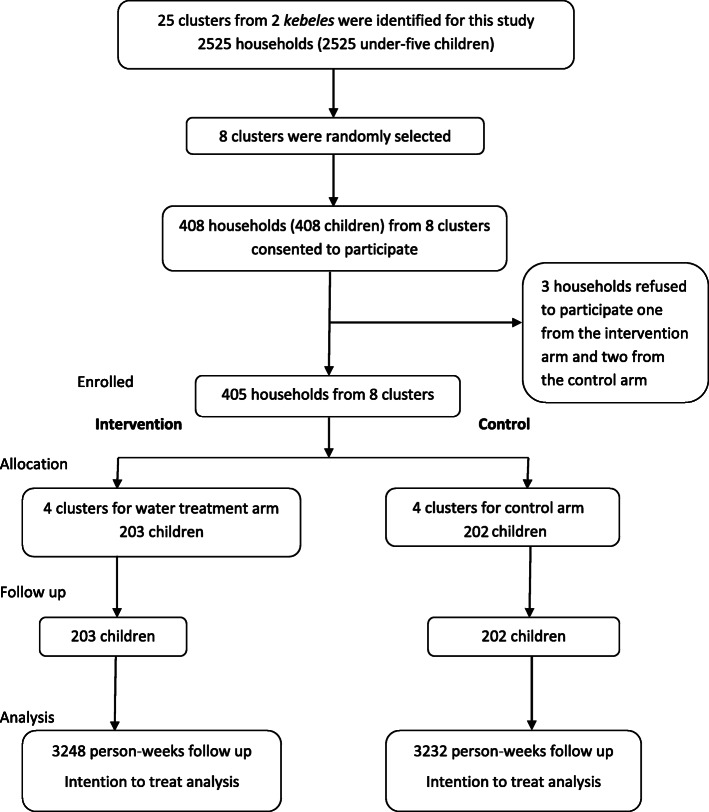
Selection of participants and the follow-up flow for the community randomized controlled trial, rural Dire Dawa, eastern Ethiopia, September 2018 through January 2019

This study used a cluster randomized controlled parallel set trial to evaluate the effectiveness of household chlorination on reducing diarrhea incidence in rural Dire Dawa from October 2018 through January 2019. There are four districts consisting of 38 *kebeles* in rural Dire Dawa. Each *kebele* was divided into sub-*kebeles* (clusters having distinct neighborhoods with defined geographical boundaries). Two districts were randomly selected. From these two districts, six *kebeles* consisting of 50 clusters were identified for this study. Of these, eight clusters from two *kebeles* were selected randomly. Households appropriate for this study had at least one under-five child. In households with more than one under-five child, the index under-five child (the child to be studied) was selected by lottery. The participant collection procedure is illustrated in Fig. 1 Selection of participants and the follow-up flow for the community randomized controlled trial, rural Dire Dawa, eastern Ethiopia, September 2018 through January 2019.

The principal investigator held a meeting with community leaders of the recruited *kebeles* to randomize clusters to the intervention arm (IA) or control arm (CA). Each cluster was given a unique identifier on a piece of papers and the papers were folded and placed into a jar. Then, an equal number of papers coded with “IA” or “CA” was placed into another jar. In front of the community leaders, two anonymous individuals (individuals who were not participating in coding) drew papers, one at a time from the two jars and draws from the two jars were matched; the drawing and matching continued until the all papers were drawn and matched. Those clusters whose unique identifiers were matched with “IA” were randomized to the intervention group and those matched with “CA” were randomized to the control group.

Cluster randomization is often advocated to minimize treatment “contamination” between intervention and control participants [27]. The intervention and control clusters in this trial were far apart, lessening the likelihood of treatment contamination between intervention and control households. Furthermore, in order to control information cross-contamination, the intervention providers were not aware of the purpose of providing the intervention materials. The study households were enrolled in April 2018 and allocated in September 2018, and the study was conducted from October 2018 through January 2019. Baseline data were collected from the two *kebeles*, 99.5% of the households in the intervention arm and 99.0% in the control arm completed the trial. The data collectors conducted a baseline survey after obtaining informed written consent from the mother or caregiver of the under-five child in each household. Finally, bottles of WaterGuard (one bottle per month), used as the water treatment intervention, were distributed at the cluster level to each household in the intervention arm.

### Cluster selection

Two of the six eligible *kebeles* were selected by a simple random sampling technique. These two *kebeles* had a total of 25 clusters. Eight clusters were selected, again by simple random sampling. The criteria for selecting clusters were as follows: they did not need to be close together; and they had to contain a minimum of 51 households with at least one under-five child. In this study, sub-*kebele* is considered as a cluster unit.

### Sample size and sampling procedure

This cluster randomized controlled trial assessed the effect of household chlorination on reduction of childhood diarrhea. In line with this, the sample size was calculated after considering 0.35 as the magnitude of the effect size. This figure means the researchers looked for a 35.0% reduction in the incidence of diarrhea in the intervention arm (receiving water, sanitation, hygiene educational messages and hand washing with soap) compared to the control arm; it was taken from a recent interventional study conducted in Jigjiga District, Somali Region [28]. Furthermore, the following were taken into consideration to come up to a calculated sample size of under-five children: 80% power, 5% significance level, 95% confidence interval, 10% contingency for non-responses, and a design effect of 4 from clustering; the calculations yielded a sample size of 204 under-five children per arm. Design effect is used as an adjustment to the sample size due to the multi-stage sampling procedure used in this trial.

**Fig. 2:**
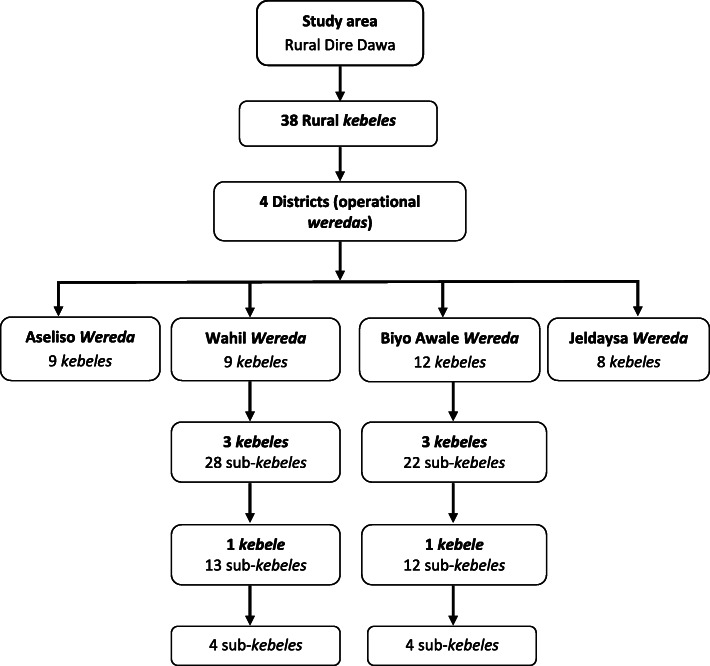
Cluster selection flow for the community randomized controlled trial, rural Dire Dawa, eastern Ethiopia, September 2018 through January 2019

To achieve the calculated sample size, a multi-stage sampling procedure was used to recruit the participants from the rural area of Dire Dawa. Two of the six eligible *kebeles* were selected by a simple random sampling technique. From these, eight of the 25 sub-*kebeles* were selected by simple random sampling. Finally, participant households were selected from “family folders” that were regularly updated by health centers and health posts again by simple random sampling (Fig. 2).

### Sample size calculation for clusters

To calculate the number of clusters required, a simple sample size calculation method for cluster randomized trials developed by Hayes and Bennett (1999) was used. For an individually randomized trial, a standard formula requires y person-weeks in each arm, where

$$ \mathrm{y}={\left({\mathrm{z}}_{\upalpha /2}+{\mathrm{z}}_{\upbeta}\right)}^2\left({\uplambda}_0+{\uplambda}_1\right)/{\left({\uplambda}_0-{\uplambda}_1\right)}^2 $$

In this formula z_α/2_ and z_β_ are standard normal distribution values corresponding to upper tail probabilities of α/2 and β, respectively. This sample size provides a power of 100(1–β)% of obtaining a significant difference (*P < α* and a two-sided test), assuming that the true (population) rates in the presence and absence of the intervention are λ_1_ and λ_0,_ respectively. After considering the following entities – z_α/2_ = 1.96, z_β_ = 0.84, λ_0_ = 10.4 [18], (i.e., incidence of diarrhea in the control arm), λ1 = 4.5 [18], (i.e., incidence of diarrhea in the intervention arm)—the calculated person-weeks (y) becomes 3.36.

For a cluster randomized trial with 3.36 person-weeks of follow up in each cluster, the number of clusters required (c) is given by the following:

$$ \mathrm{c}=1+{\left({\mathrm{z}}_{\upalpha /2}+{\mathrm{z}}_{\upbeta}\right)}^2\left[\left({\uplambda}_0+{\uplambda}_1\right)/\mathrm{y}+{\mathrm{k}}^2\left({\uplambda^2}_0+{\uplambda^2}_1\right)\right]/{\left({\uplambda}_0-{\uplambda}_1\right)}^2 $$

In this formula, k is the coefficient of variation (SD/mean) of the true rates between clusters within each arm. As a rough guideline, experience drawn from several field trials suggests that k is often ≤ 0.25 and seldom exceeds 0.5 for most health outcomes [29].

Hence, the calculated number of clusters, after considering the entities given above, became 3.53, which is approximately four clusters in each arm and eight in total. Hence four clusters were used for the intervention arm and four for the control arm.

### Intervention

Intervention providers supplied the intervention material bleach (sodium hypochlorite) to each participating household in the intervention arm (one bottle every month) for home water disinfection regularly for 16 weeks from October 2018 through January 2019. They also explained and demonstrated how to treat water using the sodium hypochlorite. The demonstration of how to make water safe using sodium hypochlorite followed CDC instructions: add one cupful of sodium hypochlorite (1.2% chlorine) to 5 gal or 20 L of water in a jerrican; cover the jerrican and shake it until the sodium hypochlorite is completely mixed with the water; wait 30 min to render the water safe to drink [30]. The concentration of chlorine present in most disinfected drinking water ranges between 0.2 and 1 mg/L [31]. The intervention providers instructed the mothers/caregivers to keep the bottle of WaterGuard out of reach of children. Intervention providers regularly checked the depletion of the WaterGuard from the bottle given to each household so the bottle could be replaced. The shelf life of the distributed WaterGuard ended December 2019. Intervention providers encouraged study participants to drink only treated water but neither encouraged nor discouraged hand washing nor other preventive actions that can decrease the occurrence of diarrhea.

### Control

In the control households, study participants were allowed to continue with their usual habits of water collection and water storage. Intervention providers neither encouraged nor discouraged drinking water treated with WaterGuard. Each participating household in the control arm was visited by data collectors once every 2 weeks to collect information about the occurrence or non-occurrence of diarrhea among under-five children.

### Measurements

In this trial, incidence of diarrhea was calculated as the number of new diarrhea episodes divided by the total person-time (i.e. person-weeks of observation) [32]. Diarrhea was defined as passage of three or more loose or liquid stools in a day [33]. An occurrence of diarrhea was considered a new episode if the child passed 3 days without symptoms of diarrhea [34].

**Table:** 

**Operational definition of terms** **Control arm:** Group of clusters provided with no household water treating product and allowed to continue with their customary practices. **Effect:** The influence of treating drinking water with a water treatment product on the incidence of diarrhea in under-five children. **Household water treatment with chlorine:** Treatment of drinking water using bleach (sodium hypochlorite) at the household level. **Improved drinking water:** Drinking water obtained from a pipe, public tap, borehole, protected spring, protected dug well, or rainwater. **Intervention arm:** Group of clusters provided with point-of-use water treatment product to treat their drinking water. **Point-of-use water treatment:** Treatment of drinking water for household use at the point-of-use.	

### Data collection

Baseline information about diarrhea-related variables such as environmental, socio-demographic and behavioral factors and two-week prevalence of diarrhea was collected using a structured questionnaire. The questionnaire was first prepared in English and then translated to the local language, Afaan Oromo, and then translated back to English to maintain consistency in the two versions. Data were collected using Afaan Oromo.

Field workers were eight data collectors, eight intervention providers, and two supervisors. The data collectors and intervention providers were local residents of their respective *kebeles.* They had completed grade 10 and speak the local language of the community. The supervisors were local residents of their respective *kebeles,* high school graduates who spoke the local language of the community. All field workers received training from the first author on techniques of interviewing and proper data collection for 2 days before the actual work. The data collection tool was pre-tested on the second day of training in a nearby *kebele* that was not included in the study and amendments were made where needed.

The main response variable of this study was diarrhea in under-five children. Data collectors collected information every 2 weeks for a period of 16 weeks, a total of eight times, according to the following parameters: occurrence of diarrhea, water treatment practices, and free residual chlorine. The secondary response variable of this study was study participants’ adherence to the intervention. The intervention material was sodium hypochlorite (a chemical compound with the formula NaOCl) distributed under the name WaterGuard, an unstable salt produced usually in aqueous solution and used as a disinfecting agent. Adherence to WaterGuard use was checked on unannounced days regularly once every 2 weeks by testing a drinking water sample for residual free chlorine using the N,N-diethyl p-phenylenediamine (DPD) colorimetric method (WAGTECH DPD1); any level greater than or equal to 0.2 mg/L was considered to be adequate adherence to treatment.

For microbiological water quality analysis, 250 ml water samples were collected at baseline and at the end of the study from drinking water storage containers of 10.0% of the participating households selected by simple random sampling. Sterile bottles were used and 1.0% sodium thiosulfate was added to the water samples from both the intervention and control arms to neutralize any chlorine present in the water. Samples were transported to Dire Dawa Water Supply and Sanitation Authority Laboratory in an ice box for processing within 4 hours of collection. Membrane filtration was used for detection and quantification of *Escherichia coli* from the water samples collected. To control the quality of the test, sterile water (negative control sample) was run with the collected water samples in the membrane filtration technique. Of the three indicator bacteria used for indication of water contamination (total coliforms, fecal thermo-tolerant coliforms, and *E. coli*), *E. coli* is regarded as the most reliable indicator of fecal contamination [35]. The membrane filter technique can be used to test relatively large numbers of samples and yields results more rapidly than the multiple fermentation tube technique [36].

### Data quality management

To ensure the quality of data, standardized tools and procedures were used. Adequate training was given to data collectors, intervention providers, and supervisors on techniques of interviewing, observation, and data recording, specific techniques for promoting drinking water treatment, and general approaches to community motivation and supervision. The expiration dates of the laboratory reagents, DPD tablets, and bottles of WaterGuard were checked. Proper drinking water treatment using sodium hypochlorite was demonstrated to intervention households by the intervention providers. Data on occurrence or non-occurrence of diarrhea, use of WaterGuard, and free residual chlorine were collected once every 2 weeks for 4 months.

Data for the intervention study were collected from 405 households for 4 months. Before data analysis, all entered data were cleaned by carrying out a frequency run procedure to identify and re-enter data missed in the original questionnaires. Water samples collected from participating households at baseline and at the end of the study were labeled using each household’s unique identifier and the results entered accordingly.

### Data analysis

Baseline and follow up visit data forms were checked for completeness and consistency before entry. The cleaned data were entered into EpiData Version 3.1 (EpiData Association, Odense, Denmark) and exported to STATA version 15.0 (StataCorp LP, College Station, TX) for analysis. All study participants were analyzed in the group to which they were randomized (i.e., by intention-to-treat analysis) in order to compare the incidence of diarrhea among children under the age of 5 years between intervention and control arms. The baseline data for intervention and control arms were analyzed and compared. Generalized estimation equation (GEE) with log link Poisson distribution family and exchangeable correlation matrix was used to compute crude incidence rate ratio and the corresponding 95% confidence intervals. GEE was also used to compute adjusted incidence rate ratio after controlling for confounding variables [37].

## Results

### Characteristics of intervention and control groups

In this trial, 204 households were assigned to the water treatment arm (intervention group) and 204 were assigned to the control arm (control group). Of these, 203 and 202 households in the intervention and control groups, respectively, completed 16 weeks of follow-up. One household from the intervention group and two households from the control groups refused to participate.

Data were collected from 405 households at baseline. The mean family size per household was 5.00. The median age of the mothers/guardians was 30 (IQR: 28–34) years and median age of the children under 5 years of age was 28 (IQR: 18–43) months. The average cluster size was 51 households with at least one under-five child. Of the under-five children, 50.4% were males and 15.6% were not breastfed. At baseline no statistically significant socio-demographic difference was observed between the intervention and control households (Table 1).

**Table 1:** Baseline demographic, environmental and socioeconomic characteristics of the study population in rural Dire Dawa, eastern Ethiopia, 2018

Variables	Control ***n*** (%)	Intervention ***n*** (%)	***P***-value
**No. of clusters**	4	4	
**No. of households**	202	203	
**No. of under-five children**	202	203	
**Mean family size per household**	5.76	4.24	
**Median age of under-five children**	38 (IQR: 32–48)	18 (IQR: 12–26)	
**Median age of mothers**	28 (IQR: 25–30)	32 (IQR: 30–36)	
**Child gender**			
Male	101 (50.0)	103 (50.7)	0.882
Female	101 (50.0)	100 (49.3)	
**Breastfeeding status**			
Not breastfed	38 (18.8)	25 (12.3)	0.071
Breastfed	164 (81.2)	178 (87.7)	
**Mother’s educational status**			
No formal education	176 (87.1)	94 (46.3)	0.000
Primary and above	26 (12.9)	109 (53.7)	
**Latrine availability**			
No	170 (84.2)	84 (41.4)	0.000
Yes	32 (15.8)	119 (58.6)	
**Availability of soap in the home**			
No	160 (79.2)	173 (85.2)	0.113
Yes	42 (20.8)	30 (14.8)	
**Refuse disposal facility available**			
No	162 (80.2)	171 (84.2)	0.288
Yes	40 (19.8)	32 (15.8)	
**Water source**			
Unimproved	6 (3.0)	56 (27.6)	0.000
Improved	196 (97.0)	147 (72.4)	
**Water storage container**			
Wide mouth	44 (21. 8)	40 (19.7)	0.606
Narrow mouth	158 (78.2)	163 (80.3)	
**Own watch**			
No	185 (91.6)	191 (94.1)	0.328
Yes	17 (8.4)	12 (5.9)	
**Own television**			
No	191 (94.6)	182 (89.7)	0.068
Yes	11 (5. 5)	21 (10.3)	
**Two week prevalence of diarrhea**			
No	153 (75.7)	153 (75.4)	0.930
Yes	49 (24.3)	50 (24.6)	

With regard to environmental sanitation characteristics, 37.3, 17.8, and 17.8% of households had a latrine, a refuse disposal facility, and soap in the home, respectively. About 15.3% households used an unimproved water source for drinking; and the majority of these (13.6%) obtaining drinking water from a stream. In 79.3% of households, the water storage container was narrow necked. Intervention and control households showed no significant difference in most of their baseline environmental characteristics (Table 1).

Prior to the intervention the two-week prevalence of diarrhea was 24.3% in the control group and 24.6% in the intervention group. With regard to economic indicators, only 7.2 and 7.9% of households possessed a watch and a television, respectively. Similarly, there were no differences between intervention and control arms with regard to diarrheal disease and socio-economic characteristics (Table 1).

### Incidence of diarrhea

Under-five children living in households that received sodium hypochlorite (bleach) evidenced fewer cases of diarrhea than children living in the control households. In the intervention households, a total of 281 cases of diarrhea were documented (8.7 cases per 100 person-weeks observation), but in the control households a total of 446 cases of diarrhea were documented (13.8 cases per 100 person-weeks observation).

In the entire study period children under the age of 5 years in the intervention arm experienced diarrhea on 1.3% of days whereas those in the control arm experienced diarrhea on 2.0% of days.

**Fig. 3:**
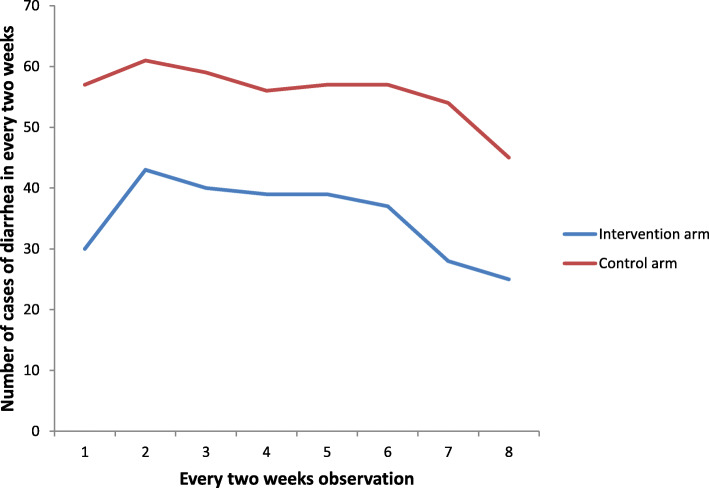
Number of episodes of diarrhea versus every two-week observation in rural Dire Dawa, eastern Ethiopia, from October 2018 through January 2019

Figure 3 illustrates diarrhea occurrence at each of the eight observation points during the 16 weeks of the study. Throughout the follow up trial, fewer children in the intervention arm were experienced diarrheal episodes than control arm.

The effect of household chlorination on reduction of childhood diarrhea differs in the different age groups of the children. The highest reduction was obtained in children aged range 1 to 2 years (42.7%), whereas the lowest reduction was observed in children aged range 3 to 4 years (30.4%) (Table 2).

**Table 2:** Number of episodes and incidence of diarrhea in control and intervention arms by age group of under-five children in rural Dire Dawa, eastern Ethiopia, from October 2018 through January 2019

Age group (years)	Control arm (***n*** = 202)			Intervention arm (***n*** = 203)			
	Number of diarrhea episodes	PWO	Diarrhea incidence (%)	Number of diarrhea episodes	PWO	Diarrhea incidence (%)	Percentage of reduction (%)
**< 1**	36	240	15.0	73	848	8.6	42.7
**1–2**	180	1328	13.6	188	2192	8.6	36.8
**3–4**	230	1664	13.8	20	208	9.6	30.4
**< 5**	446	3232	13.8	281	3248	8.7	37.0

*PWO* person-week observation, Diarrhea incidence = number of diarrhea episodes/100 person- week observation

Generalized estimating equations (GEE) with exchangeable correlation matrix and log link Poisson distribution family was employed to control for potential confounders in the multivariable analysis. Consequently, after adjusting for age of the child, gender of the child, child breastfeeding, family size, presence of refuse disposal facility, availability of latrine, availability of handwashing facility, and presence of soap in the home, under-five children in the intervention group had lower risk of diarrhea (adjusted IRR = 0.64, 95% *CI:* 0.57–0.73). A 36.0% lower incidence of diarrhea was observed in the intervention group in comparison to the control group (Table 3).

**Table 3:** Multivariable analysis of the effect of water treatment intervention on the incidence of diarrhea among children under the age of five years in rural Dire Dawa, eastern Ethiopia, from October 2018 through January 2019

Factor	Crude IRR (95% ***CI***)	Adjusted IRR (95% ***CI***)	***P***-value
**Intervention**	0.64 (0.57–0.73)	0.64 (0.57–0.73)	< 0.001
Control	1	1	
**Child age**	1.00 (0.99–1.00)	1.00 (0.99–1.00)	0.964
**Child gender**			
Female	1.00 (0.94–1.07)	1.00 (0.94–1.07)	0.961
Male	1	1	
**Breastfeeding status**			
Breastfed	0.99 (0.90–1.10)	0.99 (0.89–1.10)	0.846
Not breastfed	1	1	
**Family size**	0.99 (0.97–1.03)	0.99 (0.97–1.03)	0.910
**Presence of refuse disposal facility**			
Yes	0.99 (0.91–1.08)	0.98 (0.87–1.11)	0.808
No	1	1	
**Presence of latrine**			
Yes	1.00 (0.94–1.07)	1.01 (0.94–1.08)	0.806
No	1	1	
**Availability of handwashing facility**			
Yes	1.01 (0.85–1.20)	1.03 (0.84–1.25)	0.793
No	1	1	
**Availability of soap in the home**			
Yes	0.99 (0.90–1.08)	0.99 (0.88–1.11)	0.840
No	1	1	

*IRR* incidence rate ratio

### Drinking water quality

Drinking water was sampled for microbial testing twice from 10.0% of participating households, at the beginning and end of the study period. At the beginning of the study, 85.7% of the samples from the intervention households and 80.0% of the samples from the control households were contaminated and no significant *E. coli* difference was detected (*P* = 0.426). However, at the end of the study period, 38.1% of the samples from the interventional households and 85.0% of the samples from the control households were contaminated and a significant difference in *E. coli* counts was detected (*P* = 0.018).

Counts of *E. coli* were also compared before and after the intervention. In the intervention households, *E. coli* counts were significantly lower at post-intervention (*P* = 0.003). However, in the control households, no significant difference in *E. coli* counts was detected before intervention and after intervention (*P* = 0.692).

### Adherence to the intervention

In the intervention group, free residual chlorine was measured by the data collectors once every 2 weeks on a regular basis but on unannounced days throughout the study period. On the average, 81.3% of the drinking water samples examined had free residual chlorine of ≥ 0.2 mg/L.

At baseline, eight households (4.0%) in the control arm and 11 households (5.4%) in the intervention arm were treating their drinking water using a variety of methods (boiling, straining through clothes and, adding WaterGuard).

## Discussion

Delivery of treated and piped water to the populations in low-income countries is one of the essential United Nations Sustainable Development Goals [38]. Nevertheless, use of POU water treatments is the interim solution for people who obtain water from unimproved sources until the goal is achieved. The present study evaluated the effectiveness of household water treatment in reducing diarrhea among children under 5 years of age in rural Dire Dawa using a community-based cluster randomized controlled trial. Children in households using chlorination for their stored drinking water experienced fewer diarrheal episodes than did children in households using usual practices of water collection and storage. Point-of-use water treatment, specifically chlorination of drinking water, resulted in significantly lower (36.0%) incidence of diarrhea among children under the age of 5 years compared with children who were not given the intervention (adjusted IRR = 0.64, 95% *CI*: 0.57–0.73). This result was obtained even though the children in the intervention households were living in a highly vulnerable environment where 84.0% of households had no refuse disposal facility, 96.0% had no handwashing facility, 85.0% had no soap for washing hands, 41.0% had no latrine, 74.0% of the fathers were subsistence farmers, and 46.0% of the mothers and 30.0% of the fathers had no formal education.

In our study, 80.0% of households stored their treated drinking water in narrow-mouthed containers. Other studies reported that water in narrow-mouthed containers was less likely to be contaminated than water stored in wide-mouthed containers [39–41]. This is primarily due to the use of bowls to take water from wide mouth-containers. Diarrheal disease interventions that involve treating drinking water should therefore include the use of narrow-mouthed containers.

In our study, considerable improvement in microbial quality of drinking water was observed in the intervention households. This is in agreement with results from an analogous trial in Kersa District, eastern Ethiopia [18] and a review by Clasen and colleagues [8]. Together, these studies suggest that consistent disinfection of drinking water by chlorination prevents the water from being contaminated.

In the present study a 36.0% lower incidence of diarrhea among children under the age of 5 years who received the intervention corroborates results of trials conducted in Kenya that reported a 34.0% reduction in diarrhea [42] and Guatemala that showed a 39.0% reduction associated with water treatment [43]. On the other hand, this rate was lower than those in similar studies conducted in Bolivia (44.0%) [14], Zambia (48.0%) [15], Liberia (90.0%) [20], Pakistan (55.0%) [16], Haiti (59.0%) [17], Kersa District in Ethiopia (58.0%) [18] and Bolivia (79.0%) [19]. The difference might be explained by the fact that in our study area some of the diarrheal cases might be caused by the presence of chlorine-resistant parasitic protozoa such as oocysts of the *Cryptosporidium* species and cysts of *Giardia lamblia*.

The 11.0, 17.0 and 23.0% lower incidences of diarrhea attained in Ghana [11], Kenya [44] and Bangladesh [12], respectively, were considerably lower than that of our study (36.0%). This difference may be due to variations in study participants’ acquiescence with the intervention because, the effectiveness of household water treatment interventions at the community level may be limited by inadequate adherence [45]. Furthermore, the effectiveness of the intervention in our study was greater than in studies by Jensen et al. (2003), Colford et al. (2005), Jain et al. (2010), and Boisson et al. (2013).

These results may be due in part to our monitoring of participants’ compliance with the intervention using the DPD colorimetric test on unannounced days once every 2 weeks, giving a measured compliance of 81.3%. Our finding of compliance was consistent with results from trials in Zambia (80.5%) [15] and in Kersa District, eastern Ethiopia (79.9%) [18]. Compliance in our study was higher than in trials in Guatemala (35.0%) [13] and in Haiti (56.0%) [17], but the 85.0% compliance achieved in Liberia [20] was greater than in our study. Intermittent use of the water treatment product due to the odor and taste of sodium hypochlorite may be one reason for these variations.

In our study, the lower incidence of diarrhea (compared with non-intervention) attained in infants (42.7%) was greater than in children one to  two years (36.8%) and three to  four years (30.4%); this result is in agreement with a study carried out in Bolivia [14]. In most households in the study area, mothers usually give more attention to younger children than their older siblings likely ensuring greater use of the intervention with the younger children. Additionally, in Ethiopia most mothers boil the water they give their infants and store it in a sealed container, a practice that may reduce diarrhea transmission further in younger children [46]. Hence, the synergistic effect of chlorination, boiling, and giving greater care to young children may account for the lower incidence of diarrhea in infants than their older siblings.

The lowest reduction in incidence among children 3 to 4 years of age suggests they might have been exposed to pathogens through means of transmission other than contaminated drinking water, such as the fecal-oral route. Furthermore, children at this age actively move and play on the ground, increasing their chances of acquiring infections. Therefore, in order to reduce the occurrence of diarrhea in this age group, further intervention studies focusing on these aspects of sanitation are needed.

Among all water quality interventions, household-based chlorination is the most cost-effective [47]. In our study area, ready-made sodium hypochlorite can be purchased for USD 0.46 (15 Ethiopian Birr) per 150 ml bottle from drug vendors. This amount is enough for a rural family for approximately 1 month. Therefore, promoting regular use of the disinfectant is not only highly beneficial for the rural population, but also an affordable way to keep their children healthy. Accordingly, further research is needed to identify whether intervention households maintaining good water handling and storage practices after completion of similar projects.

There were four limitations in this study. First, we were unable to employ blinding due to the odor and taste of sodium hypochlorite. Second, we could not collect information on diarrhea on a seven-day basis. Recall bias may have occurred because information about the frequency and duration of diarrhea was collected once every 2 weeks. However, we tried to minimize the occurrence of recall bias by giving proper training to the data collectors. Third, the water treatment product (sodium hypochlorite) was provided to the intervention households free of charge; as a result, courtesy bias and the Hawthorne effect (observer effect) may have increased the effect size of the intervention. However, we tried to minimize the chances of inflated effect size by using independent intervention providers to provide the bottles of WaterGuard. Thus, the data collectors collected the data on episodes of diarrhea once every 2 weeks and had nothing to do with provision of the intervention material (WaterGuard). Fourth, some under-five children might have disliked the odor and taste of the chlorinated water and used untrusted sources, such as from neighboring households, a practice we could not monitor.

## Conclusions

In conclusion, in rural areas in Dire Dawa, water chlorination at the household level using liquid bleach (1.2% sodium hypochlorite) considerably decreased the incidence of diarrhea among children under the age of 5 years. Therefore, chlorinating drinking water at the household level may be a valuable interim solution to the problem of high rates of diarrheal disease until potable water is made accessible to the majority of the populations in Dire Dawa Administration and other Ethiopian communities. We also recommend similar interventions at the community level with the intent of assessing acceptance, expediency, and efficiency of household water treatment with chlorine solution.

## Data Availability

The datasets generated and analyzed for this study will not available publicly due to data protection law.

## Abbreviations

CA: Control Arm
CDC: Center for Disease Control and Prevention
CI: Confidence Interval
DBS: Disinfection-by-product
DPD: Diethyl para-Phenylene Diamine
GEE: Generalized Estimating Equations
HAA: Haloacetic acids
HWTS: Household Water Treatment and Safe Storage
IA: Intervention Arm
IQR: Interquartile range
IRR: Incidence rate ratio
NRERC: National Research Ethics Review Committee
PACTR: Pan African Clinical Trial Registry
POU: Point-of-use
PWO: Person Week Observation
SD: Standard Deviation
THM: Trihalomethane
WHO: World Health Organization
